# Public health program capacity for sustainability: a new framework

**DOI:** 10.1186/1748-5908-8-15

**Published:** 2013-02-01

**Authors:** Sarah F Schell, Douglas A Luke, Michael W Schooley, Michael B Elliott, Stephanie H Herbers, Nancy B Mueller, Alicia C Bunger

**Affiliations:** 1 Center for Public Health Systems Science, George Warren Brown School of Social Work, Washington University in St. Louis, 700 Rosedale Ave, Campus Box 1009, St. Louis, MO, 63112, USA; 2 Saint Louis University School of Public Health, Saint Louis University, 3545 Lafayette Ave, Room 478, St. Louis, MO, 63104, USA; 3 Insitute for Public Health, Washington University in St. Louis, 600 Euclid Avenue, Campus Box 8217, St. Louis, MO, 63110, USA; 4 Applied Research and Evaluation Branch, Division for Heart Disease and Stroke Prevention, Centers for Disease Control and Prevention, 4770 Buford Highway, Mailstop F-72, Atlanta, GA, 30341, USA; 5 The Ohio State University College of Social Work, 1947 College Road, Columbus, OH, 43210, USA

**Keywords:** Program sustainability, Concept mapping, Chronic disease programs, Evaluation, Dissemination and implementation

## Abstract

**Background:**

Public health programs can only deliver benefits if they are able to sustain activities over time. There is a broad literature on program sustainability in public health, but it is fragmented and there is a lack of consensus on core constructs. The purpose of this paper is to present a new conceptual framework for program sustainability in public health.

**Methods:**

This developmental study uses a comprehensive literature review, input from an expert panel, and the results of concept-mapping to identify the core domains of a conceptual framework for public health program capacity for sustainability. The concept-mapping process included three types of participants (scientists, funders, and practitioners) from several public health areas (*e*.*g*., tobacco control, heart disease and stroke, physical activity and nutrition, and injury prevention).

**Results:**

The literature review identified 85 relevant studies focusing on program sustainability in public health. Most of the papers described empirical studies of prevention-oriented programs aimed at the community level. The concept-mapping process identified nine core domains that affect a program’s capacity for sustainability: Political Support, Funding Stability, Partnerships, Organizational Capacity, Program Evaluation, Program Adaptation, Communications, Public Health Impacts, and Strategic Planning. Concept-mapping participants further identified 93 items across these domains that have strong face validity—89% of the individual items composing the framework had specific support in the sustainability literature.

**Conclusions:**

The sustainability framework presented here suggests that a number of selected factors may be related to a program’s ability to sustain its activities and benefits over time. These factors have been discussed in the literature, but this framework synthesizes and combines the factors and suggests how they may be interrelated with one another. The framework presents domains for public health decision makers to consider when developing and implementing prevention and intervention programs. The sustainability framework will be useful for public health decision makers, program managers, program evaluators, and dissemination and implementation researchers.

## Background

What keeps effective public health programs sustained over time? This is becoming an increasingly important question for researchers, evaluators, funders, and community partners. Public health programs focused on areas such as tobacco control and injury prevention have been shown to deliver positive health outcomes [1,2], but it is often challenging to maintain programs over long periods of time. Financial resources may only be promised from a particular funder for a short period of time, after which the program is expected to find other sources of funding. Programs may lose political and community support, or even become the targets of political or commercial opposition.

The emergence of the new discipline of dissemination and implementation science has driven a rapid increase in studies of how new scientific discoveries are translated and developed into programs, policies, and practices [3]. However, we have paid much less attention to what happens to programs once they have been implemented [4]. Programs typically need time to reach a certain level of maturity and allow health benefits to accrue. If we as a society are to get the full benefit of the significant investment in public health research and subsequent program development, we need to better understand what factors can promote long-term program sustainability.

Over time, a program ideally can sustain various elements, including its activities, community-level partnerships, organizational practices, benefits to its clients, and the salience of the program’s core issue. These are called ‘sustainability outcomes’ by Scheirer and Dearing [5], and reflect the various ways that a program can continue to have its intended effects. However, this begs the question of how a program can position itself to best ensure that these sustainability outcomes can be realized. We propose in this paper that sustainability itself is the small set of organizational and contextual factors that build the capacity for maintaining a public health program over time. That is, sustainability is the ability to maintain programming and its benefits over time.

More formally, we define sustainability capacity as the existence of structures and processes that allow a program to leverage resources to effectively implement and maintain evidence-based policies and activities. This definition is deliberately broad, and moves beyond the characteristics of the program itself that might support its sustainability to include organizational and systems characteristics. In this sense it is very similar to how others have conceptualized the ‘sustainment’ or ‘maintenance’ of public health and public service programs [6]. Sustainability capacity is a critical element of a public health program. If a program does not have sustainability capacity, it can waste money and resources, damage trust between the program and community [7], and may be limited in its ability to achieve its public health goals. Programs with a higher capacity for sustainability may be better prepared when threatened (*e*.*g*., funding cuts, infrastructure changes). Savaya *et al*. estimated that up to 40% of all new programs do not last beyond the first few years after the end of initial funding [8]. The high costs of program termination further highlights the need to understand which factors contribute to sustainability and how they can be measured and improved.

In this paper, we focus our conceptual development on the capacity of public health programs for sustainability. It is challenging to precisely define ‘public health program,’ but we follow the guidance laid out in Centers for Disease Control and Prevention (CDC) *Framework for Program Evaluation in Public Health*[9]. A public health program is any organized public health action, such as direct services, community mobilization, research, evaluation, surveillance, policy development, laboratory diagnostics, and communication campaigns. Despite this orienting definition, we developed this framework with the understanding that it might also be applied to other types of complex programs that are part of the clinical, public, or social service systems.

When a program has the necessary human, informational, and financial resources, it is more likely to achieve program goals and positively affect health [10,11]. However, little is known about the infrastructure and processes that transform these resources into positive health outcomes [12]. National guidelines such as *The Guide to Community Preventive Services*[13] and the *Cochrane Reviews*[14] identify the most effective strategies, based on current research, and guide public health professionals on what to do. But it is less clear how these evidence-based strategies are to be carried out efficiently and strategically with available resources.

Many public health agencies and foundations (*e*.*g*., CDC, Robert Wood Johnson Foundation, California Health Care Foundation, Kaiser Permanente) now require that programs provide evidence for the likelihood of program continuation, or implement tiered funding levels for multi-year grants to ensure that grantees seek new funds. There is a growing body of literature on sustainability, but neither the definition of sustainability nor the factors that affect it are well understood [15-18] Despite increasing interest, there are few evidence-based resources and no validated tools available to help public health program practitioners ensure their programs will be sustainable over time.

The purpose of this paper is to present a new conceptual framework for program sustainability capacity for public health programs. The framework was developed through a comprehensive literature review and concept-mapping process [19]. The framework was designed to be of interest to a variety of public health program stakeholders, including decision makers, practitioners, funders, researchers, and evaluators. It was also intended to be applicable to smaller (community-level) and larger (state or national-level) programs. Finally, the framework was meant to establish the basis for instrument development, so that a program’s capacity for sustainability can be better assessed in real-world public health settings.

## Methods

This is a developmental study using mixed methods to create a conceptual framework for program sustainability. Data informing the framework were collected through a literature review and an expert-informed concept-mapping process. Concept mapping is an ideal method for developing a conceptual framework. It has been used widely in public health, social, and clinical sciences for developing conceptual models, setting research and practice agendas, building logic models for program development and evaluation, and for theory development [20-23]. It has also been used recently to aid in the conceptual development of translational and implementation science [24-26].

### Literature review

The first step in the development of the sustainability conceptual framework was to identify the domains of sustainability that would be relevant for public health programs via a comprehensive literature review. A broad approach was taken, with the goal of discovering the contributors to sustainability that would be relevant for different types of public health areas (*e*.*g*., tobacco control, injury prevention), and would operate at multiple levels (*e*.*g*., state and community-level programs).

Searches were carried out by two project team members for published articles in Canada and the United States. Five electronic databases (Academic Search Premier, MedLine, CINAHL Plus, PsychINFO, and PubMed Central) were searched using a list of 17 keywords related to public health program sustainability. The article reference lists were examined for publications not captured in the original search. Finally, the terms ‘sustainability,’ ‘public health,’ and ‘program’ were searched in Google Web and Google Scholar to capture any existing grey literature. Articles included in the final set made attempts to name specific factors related to sustainability or maintenance. Those articles that mentioned sustainability without explanations of elements necessary to achieve it were excluded.

To further depict the existing literature, each article was examined for four characteristics: the health topic area, the program’s level of focus (community, state, or both), the number of sites evaluated, and the type of literature (empirical, conceptual, review, tool development, or funder report).

### Concept mapping

Concept-mapping was used to identify the conceptual structure of sustainability more precisely than was possible through review of the sustainability literature. Concept mapping is a mixed methods approach that combines qualitative group processes (*e*.*g*., brainstorming, categorizing ideas) with descriptive statistical analyses to help a group describe its ideas and represent them graphically [19]. Through this process, a visual representation of dimensions of program sustainability was created that represents the ideas of diverse representatives throughout public health.

### Approach

We followed established concept-mapping protocols [19], as described in the following eight steps.

#### Step one: create a focus prompt

A focus prompt was developed to elicit the list of ideas to be analyzed in the study. The prompt used for this study was: ‘For a public health program to successfully continue over time it needs…’ This prompt was developed by the project team in consultation with Concept Systems® trainers and an advisory committee of a small set of public health content experts.

#### Step two: develop a participant matrix to ensure representation

Using the advisory committee as the core group of informants, a snowball sampling process was used to identify experts in public health and program sustainability. These experts were included in a participant matrix to ensure that the appropriate stakeholders participated in each step of the concept-mapping process. The sample included broad expert representation from a range of public health areas, including, but not limited to: tobacco control, physical activity and nutrition, heart disease and stroke prevention, and injury prevention. Within each public health area, individuals representing research and scientific institutions, funding and advisory agencies, and state and community programs were included. The participant matrix reflected the diversity of the expert input that was used to develop the framework.

#### Step three: recruit a sample according to participant matrix

Public health experts identified in the previous step were invited via email to participate in the concept-mapping process. A total of 106 invitations were sent out to potential participants representing all of the public health content areas and job settings noted above.

#### Step four: brainstorm responses to the focus prompt

The purpose of the brainstorming is to generate a set of ideas that describe the concept of interest. An online brainstorming session was held using Concept Systems Global MAX® software. In total, 106 individuals were sent a link to the Concept Systems website and invited to brainstorm responses to the focus prompt listed in step one. The study site remained open for two weeks, and a total of 230 statements were generated. The web program does not collect information on who accesses the site for the initial item generation phase, so it is not possible to determine the number of individuals who completed this part of the exercise; this is a limitation of the present study.

#### Step five: reduce the number of items

Reducing and clarifying the generated statements is necessary to ensure success in the subsequent steps of concept mapping. The project team grouped the statements into broad themes, and eliminated or combined statements that represented the same idea. Through this process, the number of statements was reduced from 230 to 93.

#### Step six: perform sorting and rating

In order to understand how the generated statements are related to one another, participants are asked to sort them into piles based on similarity. Sixty-nine people from the original 106 brainstorming invitees were selected based on their responsiveness to the initial invitation. A total of 39 participants completed the sorting and rating phase of the concept mapping process. These 39 participants represented all four of the public health areas and all three job types from our participant matrix in step two (see Table 1). These sorts formed the basis for a concept map. Next, participants rated each statement according to scales of importance and modifiability. The instructions read as follows: ‘Please rate each statement on a 1 to 5 scale based on how important you think it is for a program to continue over time, compared to the rest,’ (1 = Not important; 2 = Somewhat important; 3 = Important; 4 = Very important; 5 = Extremely important), and ‘Now we want to know how likely it is that these items can be changed or influenced. Please rate each statement on a 1 to 5 scale in terms of how modifiable you think it is by a public health program, compared to the rest.’ (1 = Not modifiable; 2 = Somewhat modifiable; 3 = Modifiable; 4 = Very modifiable; 5 = Extremely modifiable).

**Table 1:** Concept mapping sorting and rating participant characteristics

	**Researchers/Scientists**	**Funders/Advisors**	**State/Local practitioners**	**Did not indicate**	**Total**
Tobacco Control	4	3	7		**14**
Heart Disease and Stroke	1	1	0		**2**
Physical Activity and Nutrition	7	3	5		**15**
Injury Prevention	1	4	1		**6**
Did not indicate	1			1	**2**
Total	**14**	**11**	**13**	**1**	**39**

#### Step seven: create initial concept map

A concept map was created based on the aggregated item sorts. Concept mapping creates the map using multidimensional scaling and hierarchical cluster analysis of the sorting data. Items that were sorted together by multiple concept-mapping participants are more likely to appear in the same clusters. Thus, items that were viewed as most similar by the participants end up close together on the concept map. The goal was to create a map that was simultaneously easily and quickly understood, and detailed enough to reveal useful information on sustainability. Using input from the advisory committee and project team, it was agreed that a nine-cluster solution provided the best fit to the data. The stress statistic for this concept map was .284. Stress is a measure of goodness-of-fit for the underlying multidimensional scaling solution. Previous reviews of concept mapping studies estimated an average stress value of .285, so the solution here is well in line with typical concept mapping studies [19].

#### Step eight: obtain feedback and produce final map

The grouping of the statements (*i*.*e*., content of the clusters) was then used to determine the most appropriate labels for these clusters. Feedback regarding the map was obtained from the advisory committee via a webinar, and appropriate edits were implemented. The advisory committee reviewed and approved the final statements, agreed with the final number of clusters selected, and recommended a few cluster label changes.

### Development of the capacity for sustainability framework

The face validity of the framework was established by the study team and advisory committee by examining the support for individual items in the framework, as well as for the overall structure of the framework. Three criteria were applied to each item for final inclusion in the framework: support in the literature, above-average ratings for importance, and above-average ratings for modifiability. Each framework item was given a binary code of 0 (no support found) or 1 (support found) depending on whether an analogous idea was found in the peer-reviewed sustainability literature. The project team considered it important to assess modifiability, given that a long-term goal of sustainability is a program’s continued effectiveness and the capacity to change [27].

## Results

### Literature review

**Figure 1:**
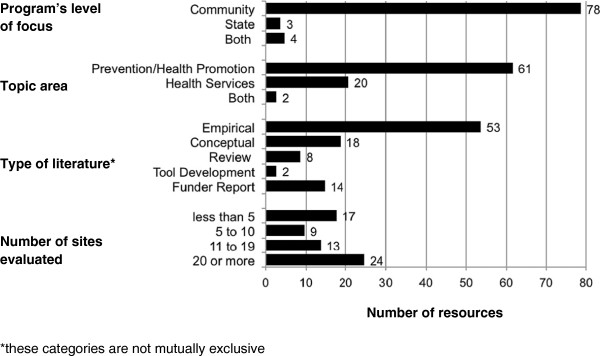
Characteristics of public health program sustainability literature (85 studies).

The current literature on sustainability spans nearly 20 years and represents an array of public health issue areas, including tobacco control, physical activity, cardiovascular health, diabetes, and asthma. We identified 85 relevant publications that encompass peer-reviewed and grey literature. Publications included empirical studies (where original data and/or analyses are presented) as well as a variety of non-empirical papers (*i*.*e*., conceptual papers, review papers, tool development, and funding agency reports). (A complete bibliography of these 85 sustainability papers is included as an Additional file 1: Appendix 1.) Figure 1 summarizes these findings.

Sustainability has been explored across many areas of public health. Over 70% of reviewed articles featured programs focusing on prevention, with most of these coming from chronic disease prevention. The other prevention programs represented a variety of topics such as substance abuse prevention [28,29], older adult health [30], and behavior change in the prevention of antibiotic resistance [31]. The remaining publications examined programs working in health service delivery or some combination of multiple categories.

The overwhelming majority of the literature examined the sustainability of community programs. There was variety in the number of program sites examined, though most publications examined 20 or more sites at which the program was being implemented. Of the peer-reviewed articles, 53 included an empirical component, 17 made some attempt at conceptualizing sustainability, and only two developed a tool to assess program sustainability. Of the tools that do exist, none have been successfully tested for reliability or validity [15,32], nor have the developed measures been retested in subsequent studies.

Most of the evidence of sustainability reported was generated by exploratory and descriptive methods. While some pieces highlighted the relevance of institutional theory [33], Schien’s work on organizational culture [34], or diffusion of innovations [35], the majority of the empirical and evaluative publications failed to draw on theory to either explain their observations or test hypotheses. There was also little consensus on definitions for major constructs such as ‘sustainability,’ ‘capacity,’ or ‘collaboration’ [36].

### Concept mapping

**Figure 2:**
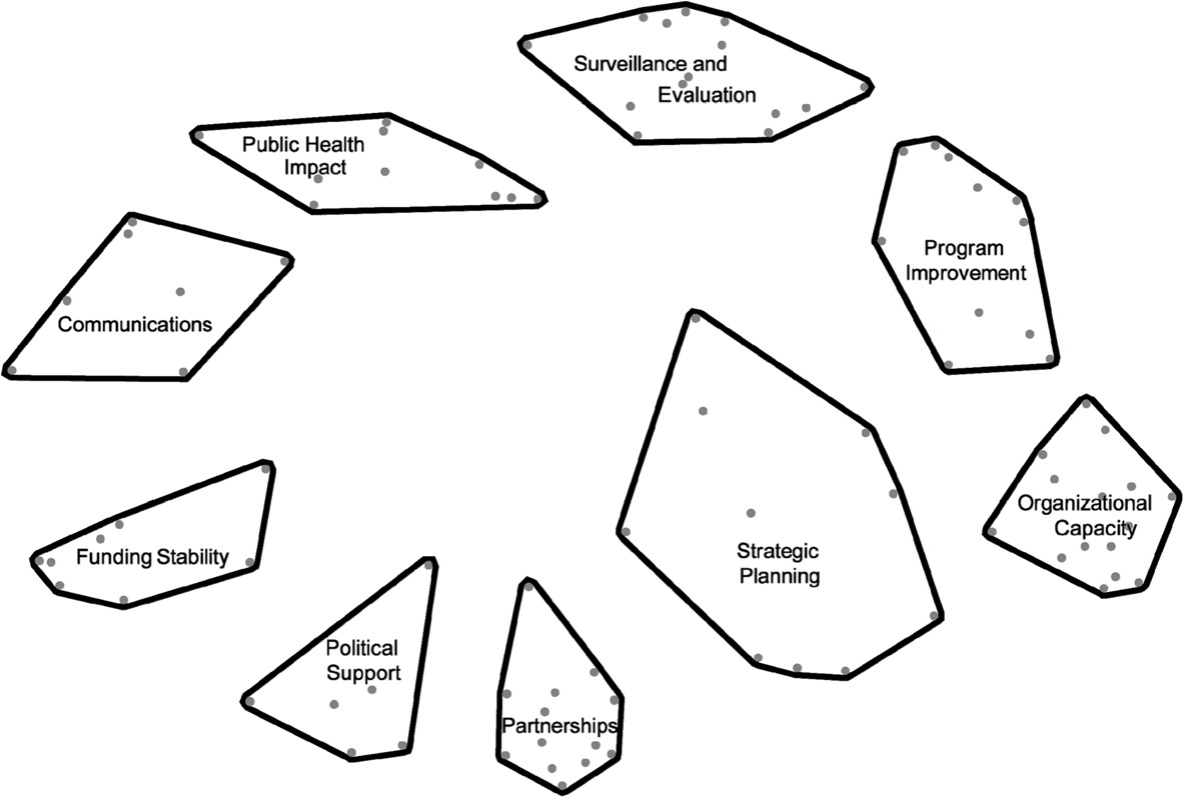
Concept map of capacity for sustainability.

Results of our concept mapping analysis identified nine domains of capacity for sustainability: *Political Support*, *Funding Stability*, *Partnerships*, *Organizational Capacity*, *Program Evaluation*, *Program Adaptation*, *Communications*, *Public Health Impacts*, and *Strategic Planning*. These domains are shown in Figure 2. The position of the domain blocks relative to each other indicates the conceptual similarities between the domains; those shown closer together are more similar than those that are farther apart. The size of each block suggests the perceived cohesiveness of the domain. The statements within smaller, tighter groupings (*e*.*g*., organizational capacity) were more conceptually cohesive than the statements within more diffuse blocks (*e*.*g*., strategic planning).

### Development of capacity for sustainability framework

Using the items that emerged from the concept mapping, we created a crosswalk with the results of our literature review. As shown in Table 2, 89% of the items in the final framework had specific support from the literature; moreover, most of these items were rated as important or very important in their domains. There was greater variability across the domains in the modifiability scores. After discussion with participants and the expert panel, we feel this reflects a recognition that program managers often have limited control over some of the aspects of program sustainability, such as external political support for their program. The combination of support from the sustainability literature and input from public health experts ensures that the framework captures the important components of sustainability capacity.

**Table 2:** Literature and concept-mapping support for framework items

**Domain**	**# of Items**	**% of Items Supported in Literature**	Average Importance Score*	Average Modifiability Score*
Political Support	6	66.7^%^	4.09	2.73
Funding Stability	8	100.0^%^	4.02	2.44
Partnerships	13	92.3^%^	3.83	3.34
Organizational Capacity	15	93.3^%^	4.03	3.31
Program Evaluation	14	78.6^%^	3.80	3.50
Program Adaptation	10	90.9^%^	3.65	3.27
Communications	7	85.7^%^	3.71	3.84
Public Health Impacts	10	100.0^%^	3.61	3.17
Strategic Planning	10	90.0^%^	3.62	3.31
**TOTAL**	**93**	**89.2**^**%**^	**3.81**	**3.24**

*Out of 5, with 5 being most important or modifiable.

**Figure 3:**
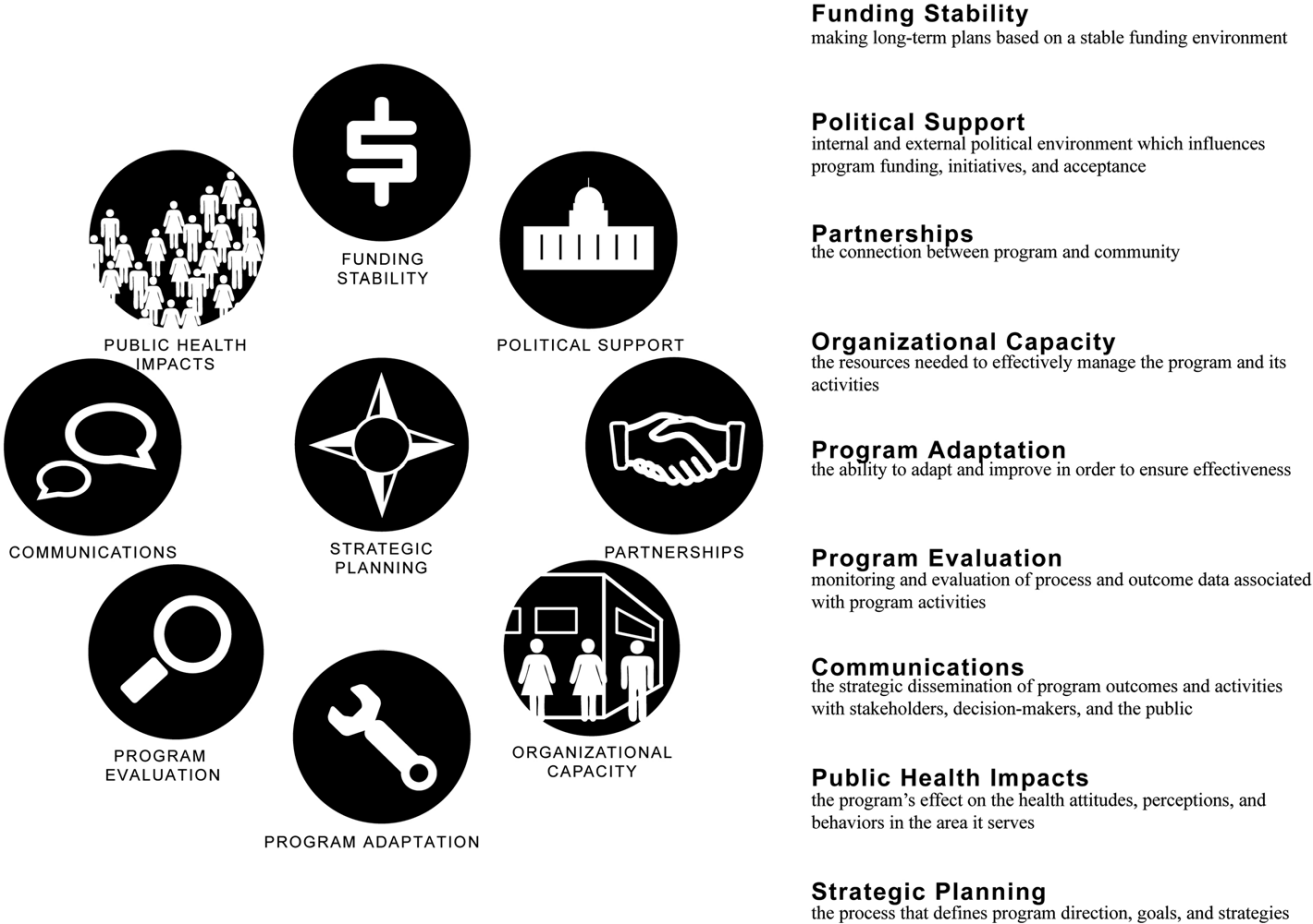
Graphic framework and definitions.

Figure 3 presents the final sustainability framework that came out of the developmental process and briefly describes each domain. For both empirical and conceptual reasons, the framework is organized in a circular pattern with strategic planning centrally positioned. Similar to the concept map, adjacent domains have more in common with one another. For example, Program Adaptation is often driven by data obtained as part of Program Evaluation activities. Furthermore, it is reasonable to apply a structural interpretation to the framework, based on the orthogonal nature of the two-dimensional domain map [37]. Specifically, the framework can be bisected diagonally to reveal an internal/external locus of control among the domains. Organizational Capacity, Program Adaptation, Program Evaluation, Communications, and Strategic Planning all involve activities that primarily occur or are managed within the program itself. Conversely, Public Health Impacts, Funding Stability, Political Support, and Partnerships are influenced by factors external to the program. This internal/external map interpretation has proven to be useful with program managers when they have worked with the framework; in particular, it helps them organize a coherent approach to program sustainability strategic planning.

## Conclusions

In this paper we present a new nine-domain framework of public health program capacity for sustainability. This framework can help establish a shared understanding of sustainability for practitioners, funders, and researchers working in a range of public health areas, and is responsive to calls for greater theoretical and definitional structure and clarity [36]. Programs that are able to sustain themselves are more likely to produce lasting outcomes and result in healthier communities [17]. By reconciling a broad but fragmented literature with an expert-informed framework, we have taken a step to aid programs in conceptualizing their capacity for sustainability.

While researchers in varied fields have studied sustainability of their specific programs, this knowledge is generally not cumulative [5,18]. Studies that have focused on a single type of program, coupled with a lack of common definitions, have prevented the field of sustainability research from moving forward [5]. The sustainability framework presented in this study is uniquely poised to promote consensus regarding definitions of sustainability for public health professionals working in a variety of substantive areas.

Previous publications on the conceptualization of sustainability have wrestled with its definition and framing: Is sustainability a process or an outcome? In a 2005 review, Scheirer makes the case that sustainability is something to be achieved [18]. However, in order to achieve sustainability, it is important to have specific program components in place. Determining the point at which a program is sustained may also prove difficult, given programs’ varying sizes, fidelity, and stage in life cycle. In addition to tracking sustained elements of a program (*i*.*e*., sustainability outcomes), it is critical to assess the characteristics of a program, its parent organization, and place in the larger service system context that lead to program sustainability (*i*.*e*., capacity for sustainability). The framework presented here explicitly focuses on sustainability capacity, as it identifies organizational and contextual characteristics that we hypothesize are necessary conditions for successfully sustaining programs over time.

This is a developmental study, and the approach we took has a number of strengths. The literature review was wide in its scope. We gathered peer-reviewed publications and grey literature from diverse topic areas within public health. Experts working in diverse public health settings informed the concept-mapping process. Concept mapping was an effective approach for engaging public health experts in diffuse geographic locations, and we were able to include expert input from more than four important public health areas (*i*.*e*., tobacco control, heart disease and stroke, physical activity and nutrition, and injury prevention). The concept-mapping methodology mixes qualitative input with statistical processes. These complementary methods provide a framework that is applicable across public health areas at the local, state, and national levels.

Despite these strengths, there are a number of important limitations in this study that some of which suggest a set of future research activities. First, concept mapping is a useful and flexible research tool, but at its core it is reliant on expert opinion. Therefore, it will be important to validate the framework to show that the various domains are all important aspects of sustainability and, more importantly, relate organizational capacity for program sustainability to sustainability outcomes such as those outlined by Scheirer and Dearing [5]. Also, although we have suggested that our capacity for sustainability framework may be applicable to programs in the clinical and social service areas, the items in our framework came from public health experts and literature. So, specific work remains to be done about determining the boundaries of this sustainability framework. Finally, the presence of many frameworks but few applicable tools suggests a need to organize the field and develop a single set of measures to assess capacity for program sustainability. Future efforts by the project team will include development of a tool based on the sustainability framework and validation of the tool across a range of public health programs. This framework grounded in the literature presents domains we believe to be critical for public health decision makers to consider when developing and implementing sustainable programs.

## Consent

Written informed consent was obtained from the participants for publication of this report and any accompanying images.

## Competing interests

All authors declare no competing interests.

## Authors’ contributions

SFS managed the concept-mapping process and wrote the first draft of the paper. DAL conceived of the study, and participated in all drafts of the paper. MWS contributed to the conceptual framework of the study and participated in the concept-mapping process. MBE assisted in the management of the concept-mapping process, and contributed to the methods and results sections of the paper. SHH provided general guidance to the study, and participated in editing of the paper. NBM developed the original plan for the sustainability study and participated in the editing process. ACB directed the literature review and participated in editing the drafts of the paper. All authors read and approved the final paper.

## Supplementary Material

Additional file 1: Appendix 1 Sustainability capacity literature review. Click here for file
