# Supplementary material for: Public health program capacity for sustainability: a new framework
Source: Implement Sci. 2013 Feb 1;8:15. doi: 10.1186/1748-5908-8-15 (PMC3599102; doi:10.1186/1748-5908-8-15)
Supplement: Additional file 1: Appendix 1 — Sustainability capacity literature review. [file 1748-5908-8-15-S1.docx]

| **Issue Area (Subarea)** | **Level of Focus** | **Type of Publication** | **Number of Sites** | **Author (Year)** | **Title** |
| --- | --- | --- | --- | --- | --- |
| Health Services | Community | Conceptual | (Conceptual) | Rosenheck R (2001) | Stages in the Implementation of Innovative Clinical Programs in Complex Organizations |
| Health Services | Community | Conceptual | (Conceptual) | Scott-Findlay S and Golden-Biddle K( 2005) | Understanding How Organizational Culture Shapes Research Use |
| Health Services | Community | Conceptual | (Conceptual) | Weiss HB, Coffman J, and Bohan-Baker M (2002) | Evaluation's Role in Supporting Initiative Sustainability |
| Health Services | Community | Empirical | 1 | Jansen M et al. (2008) | The concept of sustainability and the use of outcome indicators. A case study to continue a successful health counseling intervention |
| Health Services | Community | Empirical | 1 | Stacy D et al. (2006) | Adoption and sustainability of decision support for patients facing health decisions: an implementation case study in nursing |
| Health Services | Community | Empirical | 2 | Stetler C et al. (2009) | Institutionalizing evidence-based practice: an organizational case study using a model of strategic change |
| Health Services | Community | Empirical | 3 | Becker H, Dumas S, Houser A et al. (2000) | How Organizational Factors Contribute to Innovations in Service Delivery |
| Health Services | Community | Empirical | 4 | Goodson P, Smith MM, and Evans A et al. (2001) | Maintaining Prevention in Practice: Survival of PPIP in Primary Care Settings |
| Health Services | Community | Empirical | 5 | DeGroff A, Holden D, and Goode Green S et al. (2008) | Start-Up of the Colorectal Cancer Screening Demonstration Program |
| Health Services | Community | Empirical | 7 | Blasinsky M, Goldman H, and Unutzer J (2006) | Project IMPACT: A Report on Barriers and Facilitators to Sustainability |
| Health Services | Community | Empirical | 7 | Stange K, Goodwin M, Zyzanski S, et al. (2003) | Sustainability of a Practice-Individualized Preventive Service Delivery Intervention |
| Health Services | Community | Empirical | 13 | Bradley E, Webster T, Baker D et al. (2005) | After Adoption: Sustaining the Innovation. A Case Study of Disseminating the Hospital Elder Life Program |
| Health Services | Community | Empirical | 16 | Bowman C, Sobo J, Asch S et al (2008) | Measuring persistence of implementation: QUERI Series |
| Health Services | Community | Empirical | 18 | Steadman H, Cocozza J, Dennis D et al (2002) | Successful Program Maintenance when Federal Demonstration Dollars Stop: The ACCESS Program for Homeless Mentally Ill Persons |
| Health Services | Community | Empirical | 1091 | Leviton L, Herrera C, Pepper S et al (2006) | Faith in Action: Capacity and sustainability of volunteer organizations |
| Health Services | Community | Funder Report | 4 | Bradley E, Webster T, Baker D et al. (2004) | Translating Research into Practice: Speeding the Adoption of Innovative Health Care Programs |
| Health Services | Community | Funder Report | 215 | Kanzleiter L and Schwartz M (2010) | The Sustainability of Rural Community Health Service Providers |
| Health Services | Community | Funder Report | (Funder Report) | The Annie E Cassie Foundation (2001) | Change That Abides: A Retrospective Look at Five Community and Family Strengthening Projects, and Their Enduring Results |
| Health Services | Community | Empirical and Conceptual | 18 | Glaser E (1981) | Durability of Innovations in Human Service Interventions |
| Health Services | Community | Empirical and Conceptual | 53 | Bazzoli G, Stein R, Alexander J et al. (1997) | Public-Private Collaboration in Health and Human Service Delivery: Evidence from Community Partnerships |
| Health Services and Prevention/Health Promotion | State and Community | Conceptual | (Conceptual) | Freeman E, Presley-Cantrell L, Edwards V et al. (2010) | Garnering Partnerships to Bridge Gaps Between Mental Health, Health Care, and Public Health |
| Health Services and Prevention/Health Promotion | Community | Funder Report | (Funder Report) | Center for Civic Partnerships (2004) | Investing in Sustainability: Adding Value in Georgia |
| Prevention/Health Promotion (Asthma) | Community | Empirical | 3 | Wilson K and Kurz R (2008) | Bridging Implementation and Institutionalization Within Organizations: Proposed Employment of Continuous Quality Improvement to Further Dissemination |
| Prevention/Health Promotion (Asthma) | Community | Empirical | 7 | Friedman A and Wicklund K (2006) | Allies Against Asthma: A Midstream Comment on Sustainability |
| Prevention/Health Promotion (Asthma) | Community | Empirical | 16 | Splett P, Erickson C, Belseth S et al. ( 2006) | Evaluation and Sustainability of the Healthy Learners Asthma Initiative |
| Prevention/Health Promotion (Asthma) | Community | Empirical | 18 | Sadof M, Boschert K, Brandt S et al. (2006) | An analysis of predictors of sustainability efforts at the Inner-City Asthma Intervention sites: after the funding is gone |
| Prevention/Health Promotion (Behavior change around antibiotic resistance) | State and Community | Review | (Review) | Edgar T, Boyd S and Palamé M (2008) | Sustainability for behavior change in the fight against antibiotic resistance: a social marketing framework |
| Prevention/Health Promotion (Cancer) | Community | Empirical | 2 | Wells R, Ford E, McClure J et al (2007) | Community-Based Coalitions’ Capacity for Sustainable Action: The Role of Relationships |
| Prevention/Health Promotion (Cancer) | Community | Empirical | 320 | Glanz K, Steffen A, Elliott T et al. (2005) | Diffusion of an Effective Skin Cancer Prevention Program: Design, Theoretical Foundations, and First-Year Implementation |
| Prevention/Health Promotion (Cancer) | Community | Review | (Review) | Carpenter and Jones, Center for Collaborative Research in Health Outcomes & Policy (2006) | Charting Progress: Community-Based Colorectal Cancer Prevention Projects - Literature Review |
| Prevention/Health Promotion (Community building) | Community | Funder Report | (Funder Report) | The Annie E. Casey Foundation (2002) | End Games: The Challenge of Sustainability |
| Prevention/Health Promotion (Community health) | Community | Empirical | 11 | Trent T and Chavis D (2009) | Scope, Scale, and Sustainability: What it takes to create lasting community change |
| Prevention/Health Promotion (Diabetes) | Community | Empirical | 4 | Aitaoto N, Tsark J and Braun K (2009) | Sustainability of the Pacific Diabetes Today Coalitions |
| Prevention/Health Promotion (Diabetes) | Community | Funder Report | 14 | O’ Toole M, Brownson C, Anwuri V et al. (2009) | SUSTAINABILITY: a retrospective assessment of Diabetes Initiative Projects |
| Prevention/Health Promotion (Heart Disease) | Community | Empirical | 1 | Jackson C, Fortmann S, Flora J et al. (1994) | The capacity-building approach to intervention maintenance implemented by the Stanford Five-City Project |
| Prevention/Health Promotion (Heart Disease) | Community | Empirical | 3 | Rissel C, Finnegan J, and Bracht N (1995) | Evaluating quality and sustainability: issues and insights from the Minnesota Heart Health Program |
| Prevention/Health Promotion (Heart Disease) | Community | Empirical | 27 | Bracht N, Finnegan J, Rissel C et al. (1994) | Community ownership and program continuation following a health demonstration program |
| Prevention/Health Promotion (Heart Disease) | Community | Empirical | 42 | Riley B, Taylor S, and Elliott S (2003) | Organizational capacity and implementation change: a comparative case study of heart health promotion in Ontario public health agencies |
| Prevention/Health Promotion (Heart Disease) | Community | Empirical | 56 | Hoelscher D, Feldman H, Johnson C et al. (2004) | School-based health education programs can be maintained over time: results from the CATCH Institutionalization study |
| Prevention/Health Promotion (Heart Disease) | Community | Empirical | 96 | Osganian S , Parcel G, and Stone E (2003) | Institutionalization of a School Health Promotion Program: Background and Rationale of the Catch-on Study |
| Prevention/Health Promotion (Heart Disease) | Community | Empirical | 189 | O’Loughlin J, Renaud L, Gomez L et al. (1998) | Correlates of the Sustainability of Community-Based Heart Health Promotion Interventions |
| Prevention/Health Promotion (Heart Disease) | Community | Review and Empirical | 5 | Pluye P, Potvin L, Denis JL et al. (2005) | Program sustainability begins with the first events |
| Prevention/Health Promotion (Mental health) | Community | Conceptual | (Conceptual) | Han S and Weiss B (2005) | Sustainability of Teacher Implementation of School-Based Mental Health Programs |
| Prevention/Health Promotion (Nutrition and Substance Abuse) | Community | Empirical | 2 | Harvey G and Hurworth R (2006) | Exploring program sustainability: identifying factors in two educational initiatives in Victoria |
| Prevention/Health Promotion (Nutrition) | Community | Empirical | 1 | Elder J, Campbell N, Candelaria J et al. (1998) | Project SALSA: Development and Implementation of a Nutritional Health Promotion Project in a Latino Community |
| Prevention/Health Promotion (Nutrition) | Community | Empirical | 107 | Patterson R, Kristal A, Biener L et al. (1998) | Durability and Diffusion of the Nutrition Intervention in the Working Well Trial |
| Prevention/Health Promotion (Obesity) | Community | Empirical and Conceptual | 3 | Rudd R, Goldberg J, and Dietz W (1999) | A Five-Stage Model for Sustaining a Community Campaign |
| Prevention/Health Promotion (Older adult healht) | Community | Empirical | 20 | Evashwick C and Ory M (2003) | Organizational Characteristics of Successful Innovative Health Care Program Sustained Over Time |
| Prevention/Health Promotion (Physical Activity & Nutrition) | Community | Empirical | 487 | Seguin R, Palombo R, Economos C et al. (2008) | Factors related to leader implementation of a nationally disseminated community-based exercise program: a cross-sectional study |
| Prevention/Health Promotion (Physical Activity, Nutrition, Tobacco) | Community | Empirical | 3 | Franks A, Kedler S, Dino G et al. (2007) | School-based Programs: Lessons Learned from CATCH, Planet Health, and Not-On-Tobacco |
| Prevention/Health Promotion (Physical Activity, Nutrition, Tobacco) | Community | Empirical | 40 | Adamson K, Shepard D, Easton A et al. (2009) | The YMCA/Steps Community Collaboratives, 2004-2008 |
| Prevention/Health Promotion (Physical Activity) | Community | Empirical | 13 | Nguyen M, Gauvin L, Martineau I et al (2005) | Sustainability of the Impact of a Public Health Intervention: Lessons Learned From the Laval Walking Clubs Experience |
| Prevention/Health Promotion (Physical Activity) | Community | Empirical | 111 | Dowda M, James F, Sallis J et al (2005) | Evaluating the Sustainability of SPARK Physical Education: A Case Study of Translating Research into Practice |
| Prevention/Health Promotion (Physical Activity) | Community | Empirical | 881 | Seguin R, Economos C, Hyatt R et al. (2008) | Design and National Dissemination of the StrongWomen Community Strength Training Program |
| Prevention/Health Promotion (Substance abuse and adolescent pregnancy) | Community | Empirical | 6 | Fawcett S, Campuzano M, Paine-Andrews A (2000) | Promoting Sustainability of Community Health Initiatives: An Empirical Case Study |
| Prevention/Health Promotion (Substance Abuse) | Community | Empirical | 110 | Feinberg M, Bontempo D, and Greenberg M (2008) | Predictors and Level of Sustainability of Community Prevention Coalitions |
| Prevention/Health Promotion (Substance Abuse) | Community | Review | (Review) | Holder H and Moore R (2000) | Institutionalization of Community Action Projects to Reduce Alcohol-Use Related Problems: Systematic Facilitators |
| Prevention/Health Promotion (Substance Abuse) | Community | Review | (Review) | Pentz MA (2000) | Institutionalizing Community-Based Prevention Through Policy Change |
| Prevention/Health Promotion (Substance abuse) | State | Conceptual | (Conceptual) | Akerlund K (2000) | Prevention Program Sustainability: The State's Perspective |
| Prevention/Health Promotion (Substance Abuse) | State and Community | Conceptual | (Conceptual) | Johnson K, Hays C, Center H et al (2004) | Building capacity and sustainable prevention innovations: a sustainability planning model |
| Prevention/Health Promotion (Tobacco Control) | Community | Conceptual and Tool Development | 50 | Steckler A, Goodman R, McLeroy K et al. (1991) | Measuring the Diffusion of Innovative Health Promotion Programs |
| Prevention/Health Promotion (Tobacco Control) | Community | Empirical | 22 | Thompson B, Lichtenstein E, Corbett K et al. (2000) | Durability of tobacco control efforts in the 22 Community Intervention Trial for Smoking Cessation (COMMIT) communities 2 years after the end of intervention |
| Prevention/Health Promotion (Tobacco Control) | Community | Empirical | 77 | LaPelle N, Zapka J, and Ockene J(2006) | Sustainability of Public Health Programs: The Example of Tobacco Treatment Services in Massachusetts |
| Prevention/Health Promotion (Tobacco Control) | Community | Funder Report | 5 | The American Legacy Foundation | Sustainability Beyond Dollars: Organizations Achieving Long-Term Success in Community-Based Tobacco Control |
| Prevention/Health Promotion (Tobacco Control) | State | Empirical | 1 | Santos L, Braun K, Ae’a K et al. (2008) | Institutionalizing a Comprehensive Tobacco-Cessation Protocol in an Indigenous Health System: Lessons Learned |
| Prevention/Health Promotion (Varied) | Community | Conceptual | (Conceptual) | Hawe P, Noort M, King L et al. (1997) | Multiplying Health Gains: the critical role of capacity-building within health promotion programs |
| Prevention/Health Promotion (Varied) | Community | Empirical | 90 | Scheirer MA, Hartling G, and Hagerman D (2008) | Defining sustainability outcomes of health programs: Illustrations from an on-line survey |
| Prevention/Health Promotion (Varied) | Community | Empirical | 93 | Palm D (2006) | Designing and Building New Local Public Health in Nebraska |
| Prevention/Health Promotion (Varied) | Community | Empirical and Conceptual | 4 | Alexander J, Weiner B, Metzger M et al (2003) | Sustainability of Collaborative Capacity in Community Health Partnerships |
| Prevention/Health Promotion (Varied) | Community | Empirical and Conceptual | 10 | Goodman R and Steckler A (1989) | A model for the institutionalization of health promotion programs |
| Prevention/Health Promotion (Varied) | Community | Funder Report | 8 | The Finance Project (2002) | Sustaining Comprehensive Community Initiatives: Key Elements for Success |
| Prevention/Health Promotion (Varied) | Community | Funder Report | 13 | Venture Philanthropy Partners (2001) | Effective Capacity Building in Nonprofit Organizations |
| Prevention/Health Promotion (Varied) | Community | Funder Report | 41 | WK Kellogg Foundation (2003) | Communities Sustain Public Health Improvements Through Organized Partnership Structures |
| Prevention/Health Promotion (Varied) | Community | Funder Report | (Funder Report) | The California Wellness Foundation (2006) | Reflections - On Sustainability: Assessing the long-term impact of three TCWF Initiatives |
| Prevention/Health Promotion (Varied) | Community | Funder Report | (Funder Report) | The California Wellness Foundation (2002) | Reflections - on Sustainability |
| Prevention/Health Promotion (Varied) | Community | Funder Report | (Funder Report) | The California Wellness Foundation (2009) | Reflections - Grantmaking That Lasts: Key Findings From The Evaluation of TCWF’s Responsive Grantmaking Program |
| Prevention/Health Promotion (Varied) | Community | Funder Report | (Funder Report) | Wong E (2009) | Community Health Initiatives Sustainability Framework |
| Prevention/Health Promotion (Varied) | Community | Review | 19 | Scheirer MA (2005) | Is Sustainability Possible? A Review and Commentary on Empirical Studies of Program Sustainability |
| Prevention/Health Promotion (Varied) | Community | Review | (Review) | Roussos S and Fawcett S (2000) | A Review of Collaborative Partnerships as a Strategy for Improving Community Health |
| Prevention/Health Promotion (Varied) | Community | Review and Conceptual | (Conceptual) | Pluye P, Potvin L, and Denis JL (2004) | Making Public Health Programs Last: Conceptualizing Sustainability |
| Prevention/Health Promotion (Varied) | State | Empirical | 21 | Padgett S, Bekemeier B, and Berkowitz B (2005) | Building Sustainable Public Health Systems Change at the State Level |
| Prevention/Health Promotion (Varied) | State and Community | Conceptual | (Conceptual) | Swerissen H and Crisp B (2004) | The sustainability of health promotion interventions for different levels of social organization |
| Prevention/Health Promotion (Violence prevention) | Community | Empirical and Conceptual | 19 | Sridharan S, Go S, Zinzow H,et al. (2007) | Analysis of strategic plans to assess planning for sustainability of comprehensive community initiatives |
| Prevention/Health Promotion (Youth health) | Community | Conceptual and Tool Development | 153 | Mancini J and Marek L (2004) | Sustaining Community-Based Programs for Families: Conceptualization and Measurement |
| Prevention/Health Promotion (Youth health) | Community | Empirical | 18 | Lodl K and Stevens G (2002) | Coalition Sustainability: Long-Term Successes & Lessons Learned |
